# The impact of rotavirus vaccination on acute diarrhea in Thai children under 5 years of age in the first year of universal implementation of rotavirus vaccines in the National Immunization Program (NIP) in Thailand: a 6-year analysis

**DOI:** 10.1186/s12889-023-16958-0

**Published:** 2023-10-27

**Authors:** Busara Charoenwat, Kunanya Suwannaying, Watuhatai Paibool, Napat Laoaroon, Sumitr Sutra, Kaewjai Thepsuthammarat, Suphasarang Sirirattanakul

**Affiliations:** 1 Department of Pediatrics, Division of Gastroenterology and Hepatology, Srinagarind Hospital, Faculty of Medicine, Khon Kaen University, 123 Mitrapap Road, Muang Khon Kaen, Khon Kaen, 40002 Thailand; 2 Clinical Epidemiology Unit, Srinagarind Hospital, Faculty of Medicine, Khon Kaen University, Khon Kaen, Thailand; 3 Department of Clinical Chemistry, Faculty of Allied Health Sciences, Chulalongkorn University, Bangkok, Thailand

**Keywords:** Acute diarrhea, Rotavirus, Rotavirus vaccine, Children under 5 years of age, Children

## Abstract

**Background:**

Two types of rotavirus vaccines (RVs), Rotarix (RV1) and RotaTeq (RV5), were licensed as optional vaccines in 2012 and became part of the National Immunization Program (NIP) in the fiscal year 2020 in Thailand. The main objective was to evaluate the impact of rotavirus vaccines on the burden of acute diarrheal severity ranging from outpatient visits, diarrheal-related admission or deaths in the pre-NIP period (fiscal year 2015–2019) and in the fiscal year 2020. The minor objectives were assessed on the monthly admission rate, rotavirus vaccine coverage rate and rotavirus vaccine completed dose (RotaC).

**Methods:**

Data regarding OPD, admission, and death cases under the Thailand National Health Coverage (NHC) from fiscal year 2015–2020, which were recorded as International Classification of Diseases and Related Health Problem 10^th^ (ICD-10), were analyzed.

**Results:**

The burden of diarrheal-related disease diminished after the rotavirus vaccine was introduced in the fiscal year 2020 when compared to the previous 5 fiscal years.

The OPD visit rate decreased from 10.1 to 8.3 visits per 100 person-years (*P* < 0.001), or a 17.8% reduction (incidence rate ratio (IRR) = 0.82; 95% confidence interval (CI): 0.81 to 0.82). The admission rate significantly declined from 31.4 to 30.5 cases per 1,000 person-years, (*P* < 0.001), or a 2.9% reduction (IRR = 0.97; 95% CI: 0.96 to 0.98). The diarrheal-related mortality rate also subsided from 10.2 to 8.1 cases per 100,000 person-years (*P* 0.3), or a 20.0% reduction (IRR = 0.88; 95% CI: 0.50 to 1.22). The major population in both admissions and deaths was infants under 1 year of age (*P* < 0.001). Seasonality was seen as a constant bimodal pattern, with a significant decrease in monthly admissions after 6 months of rotavirus vaccine introduction to NIP (*P* < 0.001). RotaC was 37.4% in the first year of NIP.

**Conclusions:**

The rotavirus vaccine had a potential benefit for reducing the diarrheal disease burden, especially in infants under one year of age. Seasonality outbreaks of acute diarrhea subsided after the rotavirus vaccine was introduced. The RotaC was fairly low in the first year of the NIP. The quality of the rotavirus vaccine should be warranted.

**Trial registration:**

Number TCTR20220120003, date of registration: 20/01/2022, site: Thai Clinical Trials Registry.

## Background

Rotavirus is the most common causative agent of severe gastroenteritis in children under 5 years of age worldwide. The estimate of rotavirus gastroenteritis (RVGE) was approximately 2 million hospitalizations and 500,000 deaths of children younger than 5 years in 2000 [1, 2]. The World Health Organization (WHO) estimated RVGE-associated mortality in 2004 that was consistently as high as 527,000 deaths, and two-fifths of all deaths were in Asia [3, 4]. Although the WHO and the United Nations Children's Fund (UNICEF) launched the “WASH” policy (water, sanitation, and hand hygiene) and encouraged the initial treatment of acute diarrhea with oral rehydration salts (ORS) and zinc supplementation and promoted breastfeeding, RVGE-related hospitalization and mortality were still high. It was implied that immunization from vaccines may be a crucial part of preventive strategies for acute diarrhea [5]. In 2006, The Strategic Advisory Group of Experts on Immunization (SAGE) reviewed the data on rotavirus vaccine efficacy and safety in Americans and Europeans; therefore, SAGE was charged with advising the WHO on overall global policies to include rotavirus vaccines in the National Immunization Program (NIP), especially in Africa and Asia, which have the highest under 5-year child mortality setting, substandard WASH strategies, and high maternal HIV prevalence [6]. For Asian data, the WHO and UNICEF estimated that the RVGE-related hospitalization rate in children less than 5 years of age ranged from 2.1 to 20 cases per 1,000 person-years, and the RVGE-related death rate was 145,000 cases per year between 2000 and 2011 [7]. In 2016, 81 countries had implemented rotavirus vaccines [8], and this great impact of the vaccines, RVGE-related mortality rate in children less than 5 years of age, substantially declined to 122,000–215,000 deaths per year between 2013 and 2017, or 59% to 79% subsided from 2000 [2, 9–11]. However, only one-third of infants globally receive the rotavirus vaccine, and 80.9 million children are still unprotected [12], most of whom are Asian [8, 13]. The reasons for the struggle to implement vaccines in low-income and middle-income countries (LMICs) were the awareness of the lower effectiveness of live oral vaccines in LMICs than in developed countries [14, 15], economic problems and higher demand for other vaccines, such as the Streptococcal pneumoniae and Human Papillomavirus vaccines [16]. In Thailand, many studies have revealed the effect of rotavirus vaccines on hospitalization and death in children under 5 years of age [8, 17, 18]. The substantial study that pushed the evidence for the policy was conducted in 2010 [8], and they performed a pilot study of rotavirus vaccine effectiveness by comparing the data from 2 provinces, Sukhothai province, where an area of rotavirus vaccine was implemented, and Petchabun province, which had no rotavirus vaccine immunization. The results revealed that the rotavirus vaccine not only had a great impact on decreasing RVGE-related hospitalizations but also had an indirect “herd effect” on older children who were too old to be vaccinated. Discoveries of these effects have provided crucial evidence as policy-makers and particulate rotavirus vaccines in Thailand’s NIP. Two live attenuated oral rotavirus vaccines – monovalent rotavirus vaccine or RV1 (Rotarix, GlaxoSmithKline Biologicals, Rixensart, Belgium) and pentavalent rotavirus vaccine or RV5 (Rotateq, Merck, Whitehouse station, New Jersey, United States of America) – were documented as optional vaccines in 2012 and incorporated into the universal immunization program in the fiscal year 2020 (October 1, 2019 to September 30, 2020). In Thailand, the National Health Coverage (NCH) was realized in 2002. In 2010, the three major health schemes were the Universal Coverage Scheme (UC), the Civil Servant Medical Benefit Scheme (CSMBS) and the Social Security Scheme, which cover nearly all of the Thai population for the 23 diseases which cause the majority of the health burden in Thailand [19]. These health schemes were easily accessed and effective service system. Due to the limit of pathogen conformation tests, the most diagnoses were depended on clinical signs and symptoms. Therefore, the impact of rotavirus vaccine on RVGE or acute diarrhea in Thai children under 5 years of age is still uncertain. The main objective was to evaluate the impact of rotavirus vaccines on the burden of acute diarrheal severity ranging from mild (outpatient visits) to moderately severe as diarrheal-related admissions or deaths in the pre- NIP period and in the fiscal year 2020 as the first year of universal implementation of rotavirus vaccines in NIP. The minor objectives were to assess the impact of rotavirus vaccines on the monthly admission rate, which represented the variation according to the weather of each season, rotavirus vaccine coverage rate and RotaC in the fiscal year 2020 by utilizing the most reliable data available from the Thailand NHC data.

## Methods

A 6-year descriptive analytic study on the data regarding outpatient visit of acute diarrhea, acute diarrheal-related admissions and mortality cases by Thai children under 5 years of age in a timeframe between the fiscal years of 2015 and 2020 was carried out. Thai’s fiscal year is from October 1 to September 30 each year. The study period was divided into the “pre-NIP period” and “the first year of universal implementation of rotavirus vaccines”. The first period in which the data were in the timeframe between the fiscal years of 2015 and 2019 was the period before rotavirus vaccines were implemented in the NIP. The last dataset for the first year of the introduction of rotavirus vaccines to the universal immunization program in Thailand in the fiscal year 2020.The outpatient visit cases in children under 5 years of age represented mild symptomatic diarrheal disease, while diarrheal-related admissions or inpatient cases corresponded to moderate severity of acute diarrheal diseases that required hospitalization. “Acute diarrhea” is characterized by a decrease in stool consistency, loose or liquid stool texture and/or an increase in the frequency of bowel movements to three or more in 1 day. The duration of the disease is classified as acute onset, lasting less than 7 days; prolonged diarrhea, lasting 8 to 13 days; and chronic or persistent diarrhea, lasting 14 days or more [20]. The data were extracted from the National Health Security Office (NHSO) in Thailand which cover nearly all of the Thai population for the 23 diseases which cause the majority of the health burden in Thailand [19]. Based on the UC Scheme, which covered two-thirds of Thai residents. The outpatient visit data was derived from outpatient medical records, and diarrheal-related admissions and death information were extracted from the summary discharge of all hospitals in Thailand based on NHC data by using the International Statistical Classification of Diseases and Related Health Problems, 10^th^ Revision, Thai Modification (ICD-10-TM). Acute diarrheal diseases are defined by ICD-10-TM as intestinal infectious disease, A00-A09. The code information was analyzed for the number of outpatient visits, the rate of diarrheal-related admissions, and diarrheal-related mortality using the same ICD-10-TM code. Acute diarrheal diseases are defined by ICD-10-TM as intestinal infectious disease, A00-A09. Based on this coding, two categories were identified. First, there is a nonspecific diagnosis of infectious disease, and second, there is a more specific diagnosis of infectious diseases. A04: other bacterial intestinal infections; A05: other bacterial foodborne intoxications, not elsewhere classified; A08: viral and other specified intestinal infections; and A09: other gastroenteritis and colitis of infectious and unspecified origin were included in the first category. A00: Cholera, A01: Typhoid, A02: other Salmonella infections, A03: Shigellosis, A06: Amoebiasis, and A07: other protozoal intestinal diseases were included in the second category. A02 and A03 were subcategorized to be defined as dysenteric diarrhea. Due to the limitation that current practice does not recommend identifying all causative pathogens causing acute diarrhea in all patients. Our previous study [21] revealed two most common etiologies relating to diarrheal-related admissions and mortality in Thai children under 5 years of age between the fiscal year 2015 and 2019 were: A08: viral and other specified intestinal infections (9.8% of diarrheal-related admissions and 2.1–8.1% of diarrheal-related mortality, respectively); and A09: other gastroenteritis and colitis of infectious and unspecified origin infections (83.0% of diarrheal-related admissions and 84.1–96.8% of diarrheal-related mortality, respectively). Therefore, our present study used the two most common codes, A08 and A09, from the same data resources to represent the population of acute diarrheal cases that might be impacted by rotavirus vaccination and excluded the other causes from bacterial infection (A00: Cholera, A01: Typhoid, A02: other Salmonella infections, A03: Shigellosis, A04: other bacterial intestinal infections), bacterial food borne intoxication (A05: other bacterial foodborne intoxications, not elsewhere classified), and protozoa infection (A06: Amoebiasis, and A07: other protozoal intestinal diseases). To determinate the impact of rotavirus vaccine, the authors further analyzed the data of monthly admission rate or seasonality in the timeframe between the fiscal years of 2015 and 2020, while rotavirus vaccine coverage rate and rotavirus vaccine completed dose (RotaC) were used as the data from the fiscal year 2020 using the same ICD-10-TM code. For the rotavirus vaccine coverage rate in the first year of implementation of vaccines to NIP in the fiscal year 2020. The data were from NHSO, which is based on infants aged 2, 4, and 6 months who visited the well-baby department to administer the fundamental vaccines, Diphtheria – Tetanus – Pertussis (whole cell) – Hepatitis B – Haemophilus influenzae type b (DTwP-HB-Hib). The rotavirus vaccine coverage data were extracted for analysis. Rotavirus vaccines should receive 2 or 3 doses depending on the type of vaccine. In our practice, rotavirus vaccines were given along with the primary vaccine DTwP-HB-Hib at ages 2 and 4 months for RV1 and 2, 4, and 6 months for RV5. The first dose of rotavirus vaccine must be administered before 15 weeks of age, and the last dose must be completed before 8 months of age. The denominator used in the calculation of outpatients visit cases, diarrheal-related admission, diarrhea-related mortality rates, rotavirus vaccine coverage rate and RotaC was based on the UC scheme, in which data covered two-thirds of the Thai population. The outpatient visit rate was calculated as per 100 person-years of children aged 5 years and under, the diarrheal-related admission rate was calculated as per 1,000 person-years, and the diarrhea-related mortality rate was calculated as per 1000,000 population of the same age groups. The rotavirus vaccine coverage rate in each month was analyzed by using the standard vaccine DTwP-HB-Hib as the denominator from the UC scheme. The RotaC immunization coverage among 4-month-old infants for RV1 and 6-month-old infants for RV5 was calculated as a percentage of infants who completely received the rotavirus vaccines at the recommended doses. Data on basic demographics (age and gender), outpatient data, admission details, mortality rate were collected.

The present study was approved by the international review board, Center for Ethics in Human Research, Khon Kaen University, Human Research Ethics Committee (#HE 641526. Informed consent was waived by the Center for Ethics in Human Research, Khon Kaen University because of the lack of personally identifiable data.

### Statistical methods

Continuous and categorical variables are described as medians (interquartile range), means (standard deviation), and frequencies (%). Outpatient visits, diarrheal-related admissions, and diarrheal-related mortality rates were analyzed per 100, 1,000, and 100,000 population, respectively, of children aged 5 years and under for the same age groups. The age groups were divided into 0–1 year, > 1 to 2 years, > 2 to 3 years, > 3 to 4 years, and > 4 to 5 years of age in each year. This rate is calculated by the Poisson regression test. The monthly admission rate in each year is presented as seasonal peaks. The incidence rate ratio was analyzed to compare the incidence rate of the burden of acute diarrhea (OPD visits, admissions, and deaths) between the fiscal years 2015–2019 and 2020. The 95% confidence interval (CI) of the rate was calculated based on the normal approximation to the binomial distribution. A *P* value < 0.05 was considered statistically significant. The data were analyzed using the Stata software package, version 10.1 (StataCorp LP) program (Texas, USA).

## Results

### Outpatient visit rate

**Fig. 1:**
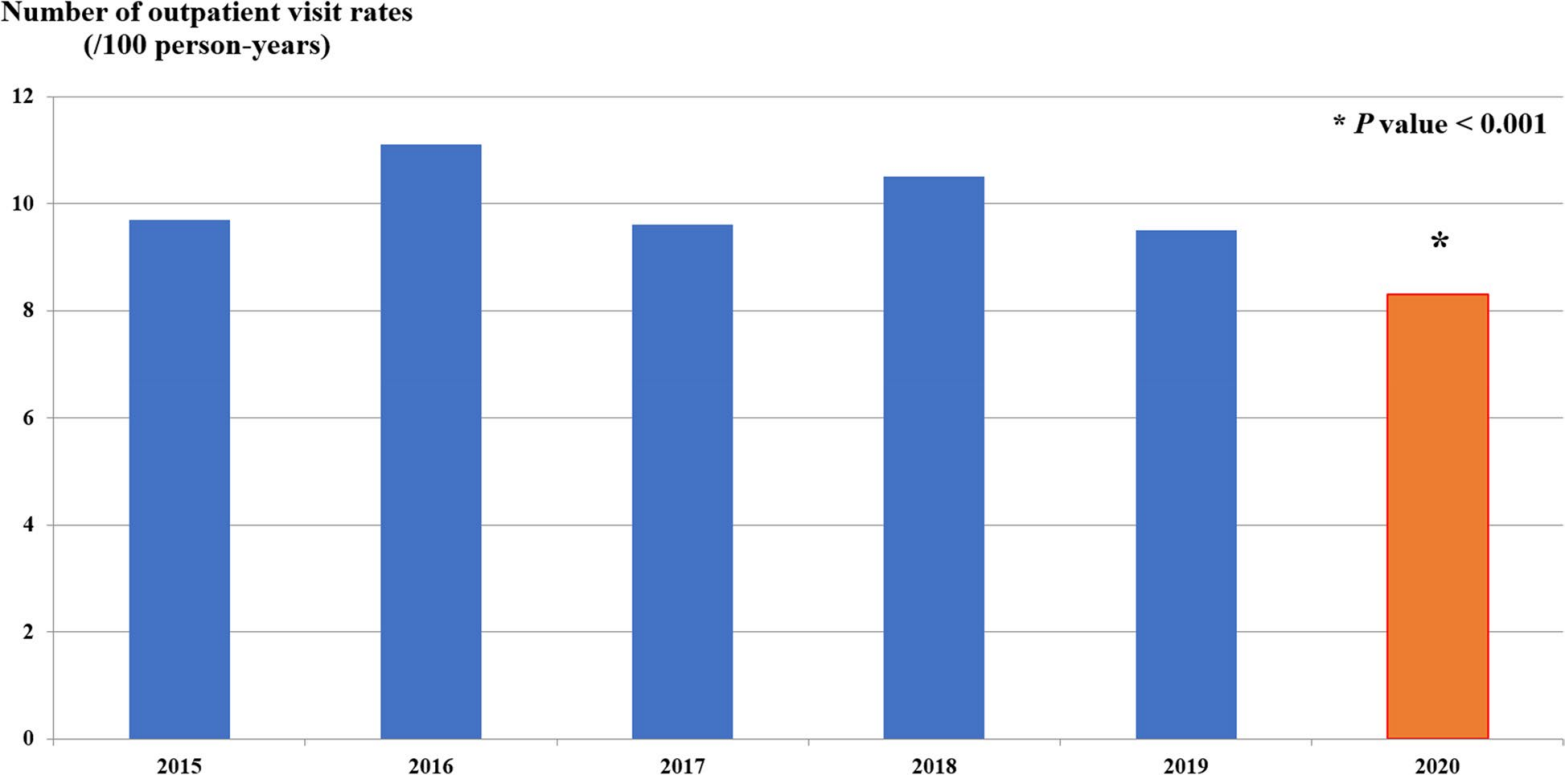
Outpatient visit rates (/100 person-years) of Thai children under 5 years of age in fiscal years 2015–2019 and 2020

For the 5-year pre-NIP period, the median outpatient visit rates due to A08: viral and other specified intestinal infections and A09: other gastroenteritis and colitis of infectious and unspecified origin were 425,504 (389,260.5–458,021.5) visits per year and decreased to 371,619 visits per year in the fiscal year 2020. There were 9.7, 11.1, 9.6, 10.5, and 9.5 visit rates per 100 person-years in 2015, 2016, 2017, 2018, and 2019, respectively, or a mean 10.1 (0.6) visit rate per 100 person-years, and a significant subsided to an 8.3 visit rate per 100 person-years in the fiscal year 2020 (*P* < 0.001) or 17.8% reduction in outpatient visit rates in the fiscal year 2020 (incidence rate ratio (IRR) = 0.82; 95% confidence interval (CI): 0.81 to 0.82) (Table 1 and Fig. 1).

**Table 1:** The incidence rate of acute diarrhea in Thai children under 5 years of age in the fiscal year of 2020 compared to the previous 5 fiscal years (2015–2019)

Type of impact	Fiscal year 2015–2019	Fiscal year 2020	IRR (95%CI)
Outpatient visit rate (/100 person-years)	10.1	8.3	0.82 (0.81, 0.82)
Diarrheal-related admissions (/1,000 person-years)	31.4	30.5	0.97 (0.96, 0.98)
Diarrheal-related mortality (/100,000 person-years)	1.0	0.81	0.80 (0.50, 1.22)

### Diarrheal-related admissions

**Fig. 2:**
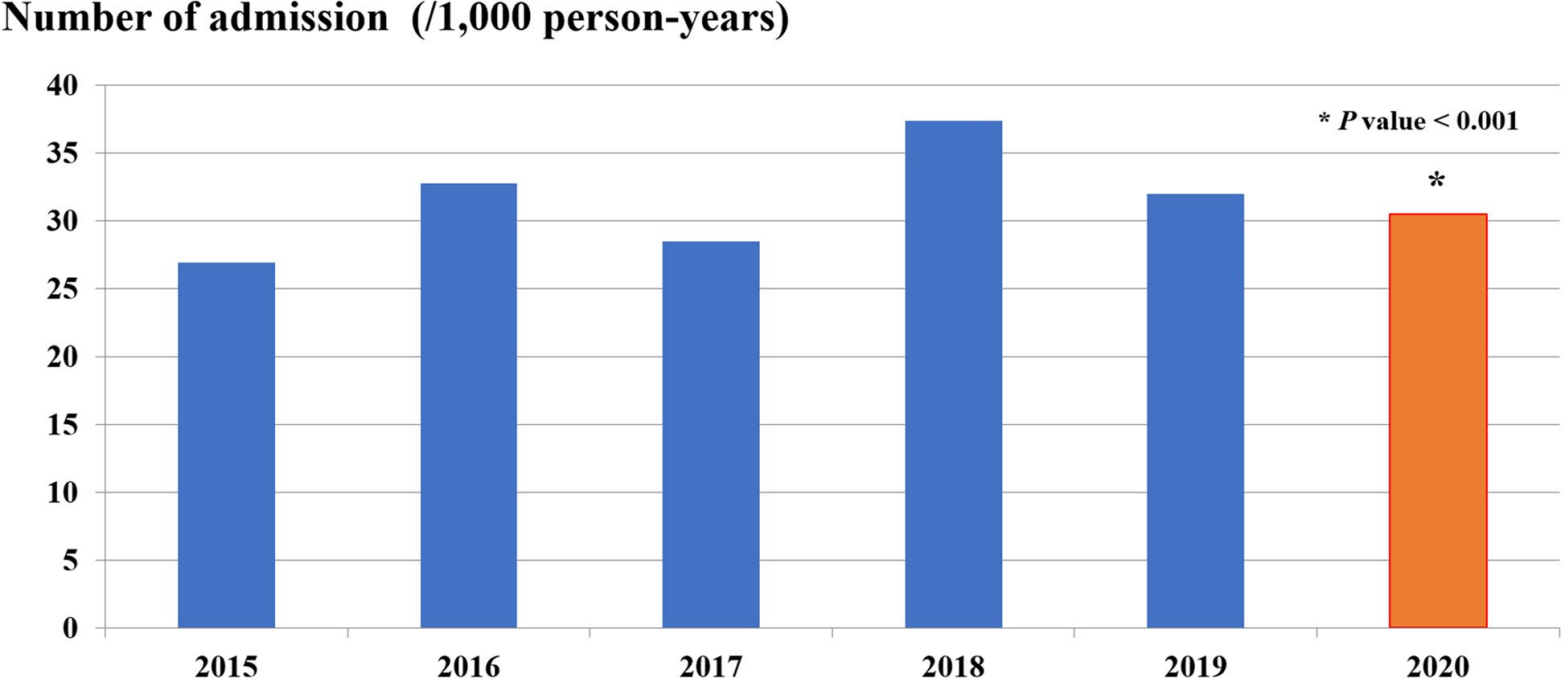
Diarrheal-related admission rates (/1,000 person-years) of Thai children under 5 years of age in fiscal years 2015–2019 and 2020

**Fig. 3:**
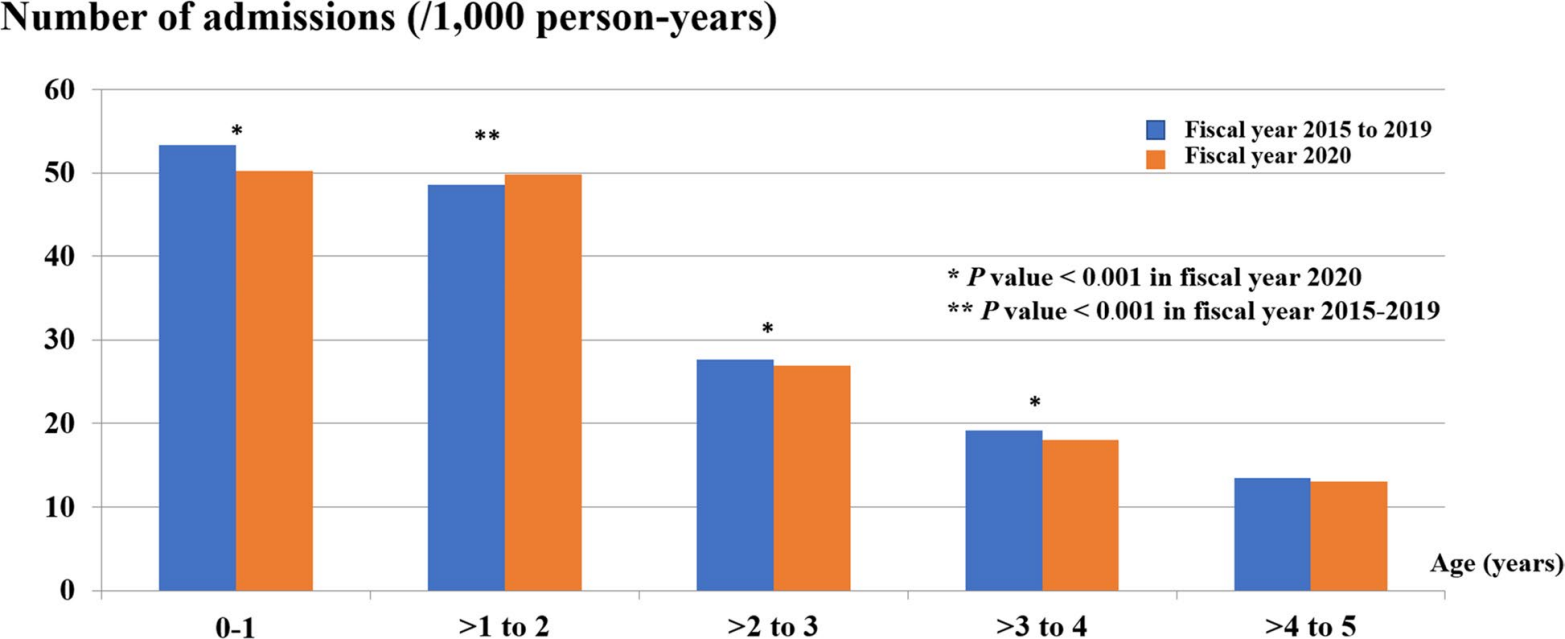
Diarrheal-related admission rates (/1,000 person-years) of Thai children under 5 years of age by age (year) in fiscal years 2015–2019 and 2020

From the data of the pre-NIP period in 5 consecutive fiscal years from 2015 to 2019 and the fiscal year 2020, the median number of diarrheal-related admissions in children 5 years of age and younger due to A08: viral and other specified intestinal infections and A09: other gastroenteritis and colitis of infectious and unspecified origin pre-NIP period was 101,996 (98,214–120,499) cases per year or a mean of 31.4 (3.7) cases per 1,000 person-years, which was significantly decreased to 93,693 cases or 30.5 cases per 1,000 person-years in the fiscal year 2020 (*P* < 0.001), or 2.9% reduction in the diarrheal-related admissions in the fiscal year 2020 (IRR = 0.97; 95% CI: 0.96 to 0.98) (Table 1 and Fig. 2). Two-thirds of admission cases in both fiscal year 2015–2019 and 2020 period were similarly infants and toddlers. For the pre-NIP period, there were 53.3 cases per 1,000 person-years (30.1%) and 48.6 cases per 1,000 person-years (29.9%) in children under 1 year of age and aged between 1 and less than 2 years, respectively. Interestingly, in the fiscal year 2020, there was a significant decrease in the admission rate to 50.2 cases per 1,000 person-years in children under 1 year of age (29%) (*P* < 0.001) but a significant increase in the admission rate to 49.8 cases per 1,000 person-years (31.2%) (*P* < 0.001) in children aged between 1 and less than 2 years. However, in the older age groups, the diarrheal-related admission rates were lower in the fiscal year 2020 (> 2 to 3 years; 27.7 versus 26.9 cases per 1,000 person-years, *P* value < 0.001, > 3 to 4 years; 19.2 versus 18.0 cases per 1,000 person-years, *P* value < 0.001, and > 4 to 5 years of age; 13.5 versus 13.1 cases per 1,000 person-years, *P* value 0.24 in the pre-NIP period and the fiscal year 2020, respectively). The age distribution at admission declined with increasing age (Fig. 3).

### Diarrheal-related mortality

**Fig. 4:**
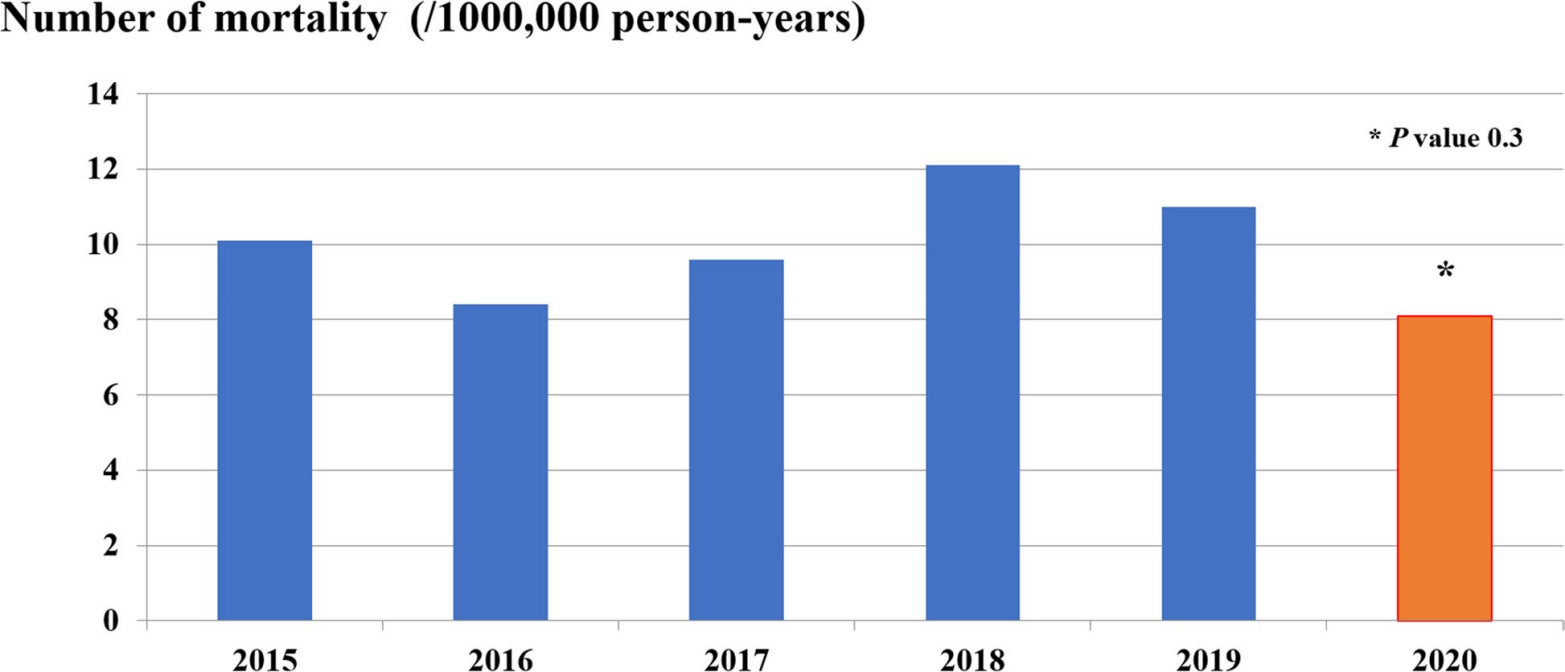
Diarrheal*-*related mortality rate (/100,000 person-years) of Thai children under 5 years in fiscal year 2015 to 2020

**Fig. 5:**
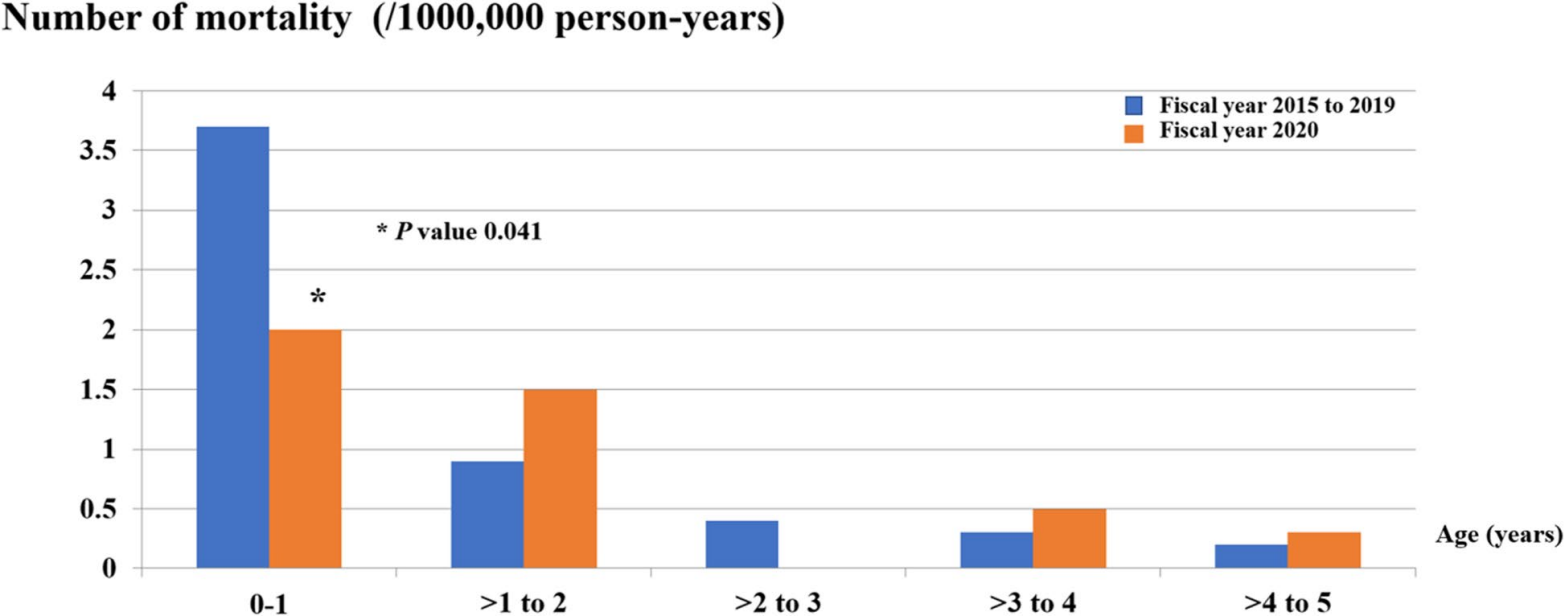
Diarrheal*-*related mortality rate (/100,000 person-years) of Thai children under 5 years of age by age (year) in fiscal years 2015–2019 and 2020

The median diarrheal-related mortality rate in children 5 years of age and younger due to A08: viral and other specified intestinal infections and A09: other gastroenteritis and colitis of infectious and unspecified origin was lower in the fiscal year 2020 than in the pre-NIP period; 25 cases per year or 0.8 cases per 100,000 person-years in the fiscal year 2020 and 35 (31.5–38.5) cases per year or a mean of 1.0 (1.2) cases per 100,000 person-years in the pre-NIP periods, respectively, *P* value 0.3, or 20.0% reduction in the diarrheal-related mortality rate in the fiscal year 2020 (IRR = 0.88; 95% CI: 0.50 to 1.22) (Table 1 and Fig. 4). Most mortality cases occurred in infants and toddlers, with the same trend as diarrheal-related admission. For children under 1 year of age, there were 3.7 cases per 100,000 person-years (65.1%) in the pre-NIP period, which significantly decreased to 2.0 cases per 1,000 person-years (44%) in the fiscal year 2020 period (*P* value 0.041). Similar findings were found in older children; there were 0.9 versus 1.5 cases per 100,000 person-years in the pre NIP period and in the fiscal year 2020, respectively, with a *P* value of 0.205 in children aged between 1 and < 2 years. In older age groups, there were 0.4 versus 0 cases per 100,000 person-years in the pre-NIP period and in the fiscal year 2020, respectively, *P* value 0.116 in > 2 to 3 years; 0.3 versus 0.5 cases per 100,000 person-years in the pre-NIP period and in the fiscal year 2020, respectively, *P* value 0.450 in > 3 to 4 years, and 0.2 versus 0.3 cases per 100,000 person-years in the pre-NIP period and in the fiscal year 2020, respectively, *P* value 0.547 in > 4 to 5 years. However, in older children aged > 2 to 5 years old, the diarrheal-related mortality rates were slightly increased without statistical significance in the fiscal year 2020. Interestingly, the diarrheal-related mortality rate in the fiscal year 2020 was significantly lower than the pre-NIP period in the same age group in children aged less than 1 year and decreased without statistical significance in those aged > 2 to 3 years. Moreover, in the older age groups, the diarrheal-related mortality rates were higher in the fiscal year 2020 in some age groups (> 3 to 4 years of age; 0.3 versus 0.5 cases per 100,000 person-years in the pre-NIP period and in the fiscal year 2020, respectively, *P* value 0.450, and > 4 to 5 years of age; 0.2 versus 0.3 cases per 100,000 person-years in the pre-NIP period and in the fiscal year 2020, respectively, *P* value 0.547). The age distribution of diarrheal-related mortality also declined with increasing age *(*Table 1 and Fig. 5*).*

### Seasonality

**Fig. 6:**
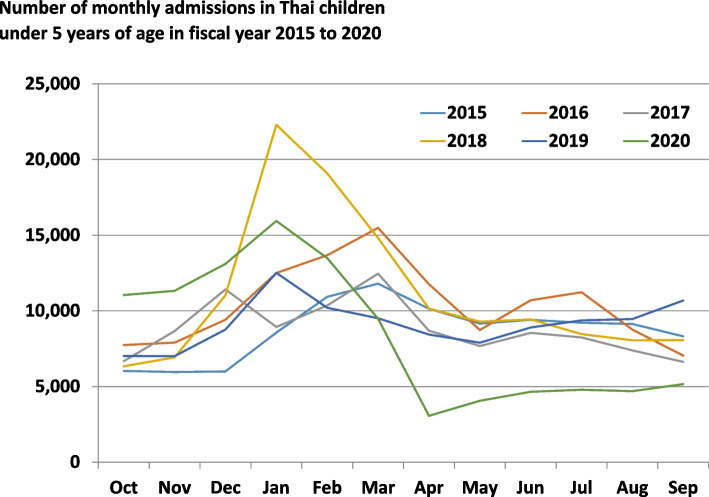
The number of monthly admissions in Thai children under 5 years of age in fiscal year 2015 to 2020

The monthly incidences of diarrheal-related admission were depicted as bimodal peaks with a constant pattern in both pre-NIP period and in the fiscal year 2020. The first higher peak was demonstrated in the cool climate, which increased in November, reached a peak in January, and then gradually declined in February. The last smaller peak was observed during early fall from April to July. A smaller peak was observed approximately 6 months after the introduction of the rotavirus vaccine to the NIP program in the fiscal year 2020 (Begin in October 2019). This peak was smaller than those of the past 5 consecutive fiscal years from 2015 to 2019, *P* value < 0.001. The incidence of monthly admissions decreased significantly in the fiscal year 2020 from March to September when compared to the five previous years during the same time periods. (IRR and 95% CI were 0.83; 95% CI: 0.80–0.85, 0.35; 95% CI: 0.33–0.37, 0.55; 95% CI: 0.53–0.58, 0.58; 95% CI: 0.55–0.60, 0.61; 95% CI: 0.59–0.64, 0.71; 95% CI: 0.68–0.74 in April, May, June, July, August, and September, respectively). A small number of acute diarrheal admission cases were found throughout the year (Fig. 6).

### Rotavirus vaccine coverage rate and RotaC in the fiscal year 2020

**Fig. 7:**
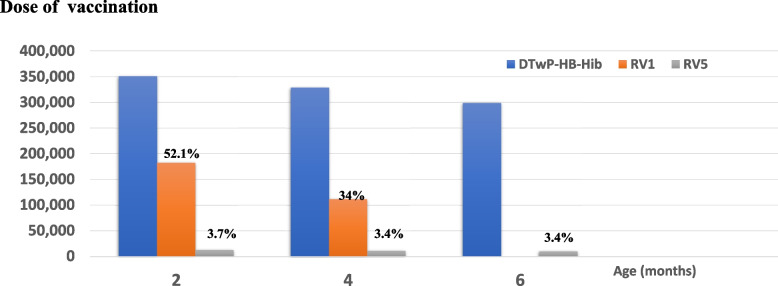
Rotavirus vaccine coverage rate in the fiscal year 2020

From the data from NHSO in the fiscal year 2020, a total of 350,843 2-month-old infants received their first dose of DTwP-HB-Hib, and 195,639 (55.8%) of them received rotavirus vaccines. A total of 182,785 infants (52.1%) received their first dose of RV1, and 12,854 infants were vaccinated with RV5 (3.7%). For 4-month-old infants, 328,583 doses of second DTwP-HB-Hib were administered, while 111,714 (34.0%) and 11,273 (3.4%) infants received the last dose of RV1 and second dose of RV5, respectively. For 6-month-old infants, 298,674 doses of the third DTwP-HB-Hib were administered, and the third or last dose of RV5 comprised 10,052 (3.4%) doses (Fig. 7). The RotaC immunization coverage among 4-month-old infants was 34% for RV1 and 3.4% for RV5 at 6 months, and the total rotavirus vaccine coverage among 6-month-old infants was 37.4%.

## Discussion

Rotavirus is a substantial cause of diarrheal disease burden, including morbidity and mortality, especially among children under 5 years of age. Over the last two decades, RVGE has resulted in approximately 25 million outpatient visits, 2 million hospitalizations, and 500,000 deaths worldwide [1–3, 22]. For Asian children’s burden of RVEG, Kawai et al. [7] revealed that RVGE-associated admission in children under 5 years old ranged from 2.1 to 20.0 cases per 100 children per year or 37.5%, with the highest rate in the southeastern area, including Thailand, and estimated 145,000 mortalities per year in the pre rotavirus vaccination era. The WHO–coordinated rotavirus surveillance network between 2008 and 2013 estimated 215,000 RVGE-related deaths worldwide, while 41% or 89,000 deaths occurred in Asia [2]. The WHO Strategic Advisory Group of Experts (SAGE) launched an earlier recommendation in 2005 on rotavirus vaccines in the United States of America and Europe, where clinical trials had proven vaccine safety and efficacy in low- and intermediate-mortality countries. Rotavirus vaccines were implemented in a national program in the United States of America in early 2006, followed by Europe and many industrialized countries [23–28]. In 2009, the WHO recommended the global use of rotavirus vaccines and weighted a priority in high mortality areas such as South, Southeastern Asia and Sub-Sahara Africa as part of both prevention (exclusive breastfeeding and WASH intervention) and treatment strategies (early rehydration with low-osmolar oral rehydration salts; ORS and zinc supplementation) [29]. In 2012, LMICs in Africa implemented rotavirus vaccines in a routine immunization program [30–33]. Surprisingly, 7 years after the WHO recommendation, up to 40% of Asian populations were unvaccinated. The delayed implementation of rotavirus vaccines was concerned with the lower efficacy of live oral vaccines in low-income countries, financial issues and the cost of other new vaccines in the NIP [13–16]. Strategies to drive rotavirus vaccines to NIP were initiated. A 2-year rotavirus vaccine sentinel surveillance project was performed [34]. They calculated the RVGE-associated admission rate, which was 11.28 admissions per 1,000 population, and one-half were infants younger than 1 year old. In 2011, an observational study to evaluate the benefit of the first introduction of rotavirus vaccines in Thailand was conducted [8], and they compared the data from Sukhothai province as a vaccinated area with Petchabun province as an unvaccinated area. The key result of this study was vaccine efficacy on RVGE-associated admission rate (88%, 95% CI: 76%-94%) and indirect herd immunity among older children who were too old to be vaccinated. Then, in 2012, RV1 and RV5 were licensed as optional vaccines and incorporated into the NIP in 2020. In Thailand, the rotavirus vaccine was administered simultaneously with DTwP-HB-Hib at 2 and 4 months of age for RV1 and at 2, 4 and 6 months of age for RV5. The coadministration of rotavirus vaccine with other vaccines did not intervene in immunogenicity, efficacy, or safety [35]. As our country used both RV1 and RV5, switching from one to another was a concern. Because the best recommendation was to complete the same rotavirus vaccine series, in the case of incomplete vaccination, a complete series with another rotavirus vaccine was suggested. A study in the United States of America found that 3 dose series of mixed RV1 and RV5 were also tolerated and induced immunogenicity compared to those who received a series of single vaccine types [36]. The effectiveness of complete series regardless of how many types of vaccine product is greater than incomplete the series with one product [29].

Many industrialized countries report high rotavirus vaccine efficacy and effectiveness on RVGE-related hospitalization and mortality. However, Thai’s current practice policy does not recommend identifying all pathogens causing acute diarrhea in all cases [19]. Therefore, the vaccine impact is the most appropriate marker of evidence of the rotavirus vaccine’s benefits in our setting.

The data on rotavirus-associated outpatient visits were limited due to underestimated or incomplete medical records in the ICD-10-TM system. However, the rotavirus-associated outpatient visit rate was a crucial part of evaluating the impact of the rotavirus vaccine on the burden of acute diarrhea. Parashar et al. [1] estimated the rotavirus-associated outpatient visits among children under 5 years of age, and there were 202, 217, and 3332 visits per 1,000 live births by age 5 years for low-, middle-, and high-income countries, respectively. A systematic review of the burden of RVGE in Asia [7] that comprised East and Central Asia and included upper- and low- to middle-income countries revealed that the mean rotavirus-associated outpatient visits in children under 5 years of age in the pre-NIP period between 1999 and 2009 was 25.7 (13.8) visits per 1,000 children per year. Their outpatient visit rate was lower than that in our study, with a mean of 10.1 visits per 100 person-years in the pre-NIP period. However, the heterogeneity of their study was not included, and the variety of sanitation systems and income levels in different countries might have affected the results. Two studies in Finland evaluated the impact of rotavirus vaccine by restrictive use of RV5 on rotavirus-associated outpatient visits 2 and 5 years after universal RV5 vaccination. They detected a significant reduction in the mean rotavirus-associated outpatient visit rate of 88% (*p* < 0.001) in the post-NIP 2-year period in 2009–2011 and up to 91% (*p* < 0.001) in the post-NIP 5-year period in 2009–2014 when compared to the reference pre-NIP 2-year period in 200–2008. The reduction rate was similar to that in previous studies in the United States of America [37–39]. In Thailand, two available RV1 and RV5 were licensed. Our study revealed a significant reduction in the diarrheal-associated outpatient visit rate in the fiscal year 2020 of approximately 1.8 visits per 100 person-years, *P* value 0.3, or 20.0% reduction in the diarrheal-related mortality rate in the fiscal year 2020 (IRR = 0.88; 95% CI: 0.50 to 1.22) which was as high as the reduction in the outpatient visit rate in high-income countries. Although there is concern about the efficacy of live oral vaccines in LMICs, the high first-dose coverage in our country (55%), which was near the 58% first-dose coverage rate in the United States of America in 2007 [40], might have a positive effect on reducing the outpatient visit rate.

Based on NHC data during the pre-NIP and the fiscal year 2020, the mean diarrheal-related admission rate was 31.4 cases per 1,000 person-years in the pre-NIP period, whereas in the fiscal year 2020, the mean diarrheal-related admission rate was significantly decreased to 30.5 cases per 1,000 person-years, *p* value < 0.001, or 2.9% reduction in the diarrheal-related admissions in the fiscal year 2020 (IRR = 0.97; 95% CI: 0.96 to 0.98). In Thailand, the crucial pilot research as a policy marker to drive rotavirus vaccine into NIP evaluated the first introduction of rotavirus vaccine revealed vaccine effectiveness for RVGE-associated admission rate was 88% (95% CI: 76%-94%) [8]. In a global setting, data from the WHO coordinated Global Rotavirus Surveillance Network (GRSN) from 2008 to 2016 revealed a relative decline in RVGE-associated diarrhea in children under 5 years of age from 39.6% (95% CI: 35.4%-43.8%) to 23.0% (95% CI: 0.7%-57.7%) or a 40% reduction after the rotavirus vaccine was introduced [41]. From Asia’s data but not including Thailand, the estimate of rotavirus vaccine impact if all 43 Asian countries were vaccine coverage, RVGE-related admission rate will subside for 40% from present baseline, but in the present situation that only 8 Asian countries were vaccine coverage [13]. The systemic review of vaccine impact in industrialized countries between 2006 and 2010 reported a significant reduction in RVGE-associated admission since the rotavirus vaccine was implemented [42]. Several factors affecting rotavirus vaccine performance should be considered in our middle income [43] and medium mortality strata setting of children under 5 years of age [44]. A Cochran review stated that vaccine efficacy against severe RVGE was better in low-mortality strata than in high-mortality strata [45] and suboptimal protection effect of rotavirus vaccine in LMICs [5]. However, the mechanisms remain incompletely clear, and the multifactorial might be explained, including the high rate of infection and impaired immunogenicity, for example coinfection, maternal antibodies, environmental enteropathy, nutritional deficiency, gut dysbiosis and some genetic factors [46–50]. From this knowledge, the vaccine impact and factors that might underperform rotavirus vaccine in our setting should continue to be studied for a longer period. For age distribution, our findings showed that there was a significant reduction in the diarrheal-related admission rate in infants under 1 year of age pre-NIP period and in the fiscal year 2020, *p* < 0.001. These data might be strong evidence of the direct effect of a vaccine on decreasing RVGE-associated admission in children of vaccinated age (less than 1 year old). Apart from the direct effect of the vaccine, we also observed that age shifted upward in older children in age Group 1 to ≤ 2 years in the pre-NIP period and in the fiscal year 2020 (48.6 cases per 1,000 person-years and 49.8 cases per 1,000 person- pre-NIP period and in the fiscal year 2020, respectively, *p* < 0.001). Similar results were described by a study in developed countries [51–53]. The age shift in the case of diarrheal-related admission was reflected in a protective effect after vaccination [41]. Moreover, the proportion of all diarrheal-related admissions among older children who were not eligible for vaccination also decreased in all age groups (2 to < 3, 3 to < 4, and 4 to ≤ 5 years). These important data support the evidence of an indirect effect or herd immunity of the rotavirus vaccine. The herd effect is probably interrupted by household transmission among children [54, 55]. Although herd immunity is infrequently reported in LMICs [56], it is present in our literature.

Before the rotavirus vaccine was available in 2006, RVGE had a vast majority on the burden of acute diarrhea in children under 5 years old, with an estimated more than 500,000 deaths and 2 million hospitalizations globally in 2000 [1, 2]. In Thailand, Jiraphongsa et al. [34] estimated RVGE-associated death resulting in 2.2 deaths per 100,000 children per year in 2008. In the current situation in which rotavirus vaccines have become available, RVGE also causes a burden in small children. However, overall RVGE-associated admission and death declined. From 2013 to 2017, the estimate of RVGE-associated mortality subsided 59–75% since 2002 [2, 9–11]. Another study [9] based on population in 2013 compared the estimate of RVGE-associated mortality in children under 5 years old from the Child Health Epidemiology Reference Group of the World Health Organization (WHO) and UNICEF (CHERG), the Global Burden of Disease Study (GBD) and the WHO and Centers for Disease Control and Prevention (WHO/CDC), the three largest health organizations. The global estimated RVGE-associated mortality was 157,398, 122,322, and 215,757 deaths per year from CHERG, GBD, and WHO/CDC, respectively. Furthermore, they analyzed and revealed that acute diarrhea was associated with diarrheal-related death in children under 5 years of age by approximately 65% (95% CI: 57%-74%). They reanalyzed data from the Global Enteric Multicenter Study (GEMS) and found that 28% of children under 5 years of age were rotavirus-positive for acute diarrheal death. From an Asian perspective, Burnett et al. [13] estimated the impact of the rotavirus vaccine on RVGE-associated death, and the results revealed that the rotavirus vaccine decreased death by approximately 40% (95% CI: 35%-46%) or from 88,890 (95% CI: 84,014–93,478) to 35,865 (95% CI: 30,427–41,370) annual RVGE-associated deaths. Our result was similar to the overall results. The diarrheal-associated mortality decreased from 1.2 to 0.8 per 100,000 person-years pre-NIP period and in the fiscal year 2020, respectively*, P* value 0.3, or 20.0% reduction in the diarrheal-related mortality rate in the fiscal year 2020 (IRR = 0.88; 95% CI: 0.50 to 1.22). The majority of diarrheal-associated deaths were observed in children under 1 year of age. However, death was significantly decreased from 3.7 to 2.0 per 100,000 person-years pre- and post-NIP, respectively *(P* value 0.04). Similar to a study in 2010 [57] and our previous study [21], the highest diarrheal-associated mortality was discovered in the first year of life and then gradually subsided with increasing age. The reason could be attributed to the immature immune system along with personal hygiene [58–60].

Studies in Asia have shown a variety of seasonal patterns that depend on terrain and atmospheric conditions. Kawai et al. [7] reviewed the various seasonal variations in RVGEs in Asia. There were three patterns: the peak in the cool period in many temperate countries in Eastern and Central Asia, two district seasonal peaks in the winter and monsoon season in tropical areas such as Southeast and Southern Asia, and some landscapes, including Thailand, where RVGEs were detected year-round. Even in studies in Thailand, some studies stated that RVGEs were detected year-round [17, 61], while others detected bimodal peaks in the cool and rainy seasons [21, 57, 61, 62]. In this study, the result of the monthly admission or seasonal pattern was demonstrated as a constant pattern with consistency over both pre-NIP period and in the fiscal year 2020. The highest peak in cool temperature was followed by a smaller peak in the wet season, A smaller peak was lower than those of the past 5 consecutive fiscal years from 2015 to 2019, *P* value < 0.001. The incidence of monthly admissions decreased significantly in the fiscal year 2020 from March to September when compared to the five previous years during the same time periods. This might demonstrate the positive effect of the rotavirus vaccine on diarrhea burden. A study in the first year after the introduction of RV5 in the United States of America in 2007–2008 [38] revealed a delay in the rotavirus season lasting 14 weeks and a lower magnitude of the peak compared with the pre-vaccine season from 2000 to 2006. One study in Latin America [63] from 2004 to 2017 also had the same trend of a later shift and smaller rotavirus season. These were the results of the direct immunity effect of the rotavirus vaccine to decrease severe RVGE and indirect or herd immunity to protected unvaccinated individuals. Long-term follow-up in the duration of rotavirus implementation and RotaC might be beneficial for the seasonal pattern in Thailand.

After the WHO launched the rotavirus vaccine policy in 2006, a year later in 2007, as the first year of rotavirus vaccine introduction in the United States of America, data from 6 sentinel sites demonstrated that first-dose rotavirus vaccine coverage among 3-month-old infants reached half of the infant population (58%; range 51%-68%) [40]. For the European site, the rotavirus vaccine was introduced to Finland in 2006, and the vaccine coverage rate rose from 0 to 30% between 2006 and 2008 and then sharply increased to 95%-97% in 2009. In Thailand’s practice, rotavirus vaccines were coadministered with DTwP-HB-Hib, and rotavirus vaccine coverage was based on the DTwP-HB-Hib population as a denominator. Demographic and Health Survey (DHS) [64] estimated rotavirus vaccine coverage in LMICs using a similar method as the WHO-UNICEF estimated for DTP vaccine coverage. For the first year of rotavirus vaccine in national immunization, the first-dose rotavirus vaccine coverage was 55.8%, while the second-dose rotavirus vaccine coverage was 37.4%. However, the RotaC was 37.4%, so for more data, a longer period should be used.

This study has some limitations. First, this retrospective fashion depended on ICD-10 TM codes, so sometimes inaccurate and incomplete medical records were obtained. Moreover, Thai’s current practical management and clinical practice guidelines for acute diarrhea do not suggest identifying the causative pathogens in all cases, which causes vaccine efficacy and vaccine effectiveness to not be performed. Finally, the duration of NIP was only 1 year, and a longer duration of post-NIP should be followed.

## Conclusions

The implementation of the rotavirus vaccine into the NIP in the fiscal year 2020 had the potential benefit of a major reduction in the diarrheal disease burden of all outpatient visits, hospitalizations, and mortality. Seasonality outbreaks of acute diarrhea subsided after 6 months when the rotavirus vaccine was introduced. The RotaC was fairly low in the first year of the NIP. Long-term follow-up of vaccine impact, vaccine efficacy, vaccine effectiveness, and factors associated with rotavirus vaccine quality is warranted.

## Data Availability

The data that support the findings of this study are available from Thailand National Health Coverage but restrictions apply to the availability of these data, which were used under license for the current study, and so are not publicly available. Data are however available from the authors upon reasonable request and with permission of Thailand National Health Coverage.

## Abbreviations

CHERG: Child Health Epidemiology Reference Group of the World Health Organization (WHO) and UNICEF
CSMBS: Civil Servant Medical Benefit Scheme
DHS: Demographic and Health Survey
DTwP-HB-Hib: Diphtheria – Tetanus – Pertussis (whole cell) – Hepatitis B – Haemophilus influenzae type b
GEMS: Global Enteric Multicenter Study
GBD: Global Burden of Disease Study
GRSN: Global Rotavirus Surveillance Network
NCH: National Health Coverage
NHSO: National Health Society Office
NIP: The National Immunization Program
ORS: Oral rehydration salts
LMICs: Low—income and middle—income countries
RotaC: Rotavirus vaccines completed dose
RVGE: Rotavirus gastroenteritis
RV1: Monovalent rotavirus vaccine
RV5: Pentavalent rotavirus vaccine
SAGE: Strategic Advisory Group of Experts on Immunization
UC: Universal Coverage Scheme
UNICEF: United Nations Children's Fund
WHO: World Health Organization
WHO/CDC: The WHO and Centers for Disease Control and Prevention
WASH: Water, sanitation, and hand hygiene
